# Pulmonary Aspergillosis and Low HIES Score in a Family with STAT3 N-Terminal Domain Mutation

**DOI:** 10.1007/s10875-025-01867-1

**Published:** 2025-02-10

**Authors:** Suiane Lima de Souza, Takaki Asano, Virpi Glumoff, Salla Keskitalo, Keela Pikkarainen, Timi Martelius, Meri Kaustio, Janna Saarela, Outi Kuismin, Elisa Lappi-Blanco, Airi Jartti, Fredrik Yannopoulos, Leena Tiitto, Mikko R. J. Seppänen, Bertrand Boisson, Jean-Laurent Casanova, Markku Varjosalo, Timo Hautala, Zhi Chen

**Affiliations:** 1 Faculty of Biochemistry and Molecular Medicine, University of Oulu, Aapistie 7, Oulu, Finland; 2 St. Giles Laboratory of Human Genetics of Infectious Diseases, Rockefeller Branch, The Rockefeller University, New York, NY 10065 USA; 3 Research Unit of Internal Medicine and Biomedicine, University of Oulu, Aapistie 5, Oulu, Finland; 4 Molecular Systems Biology Group, Institute of Biotechnology, University of Helsinki, Helsinki, Finland; 5 Inflammation Center, Infectious Diseases, HUS Helsinki University Hospital and University of Helsinki, Helsinki, Finland; 6 Institute for Molecular Medicine Finland HiLIFE, University of Helsinki, Helsinki, Finland; 7 Centre for Molecular Medicine Norway, University of Oslo, Oslo, Norway; 8 Department of Clinical Genetics, Oulu University Hospital, Kajaanintie 50, 90220 Oulu, Finland; 9 Department of Pathology, University of Oulu, Aapistie 5, Oulu, Finland; 10 Department of Radiology, Oulu University Hospital, Kajaanintie 50, 90220 Oulu, Finland; 11 Department of Cardiothoracic Surgery, Oulu University Hospital, Oulu, Finland; 12 Pulmonary Unit, Department of Medicine, Oulu University Hospital, University of Oulu, 90220 Oulu, Finland; 13 Translational Immunology Research Program, University of Helsinki, Helsinki, Finland; 14 Pediatric Research Center New Children’s Hospital, University of Helsinki and, HUS Helsinki University Hospital, Helsinki, Finland; 15 Rare Diseases Center and Pediatric Research Center New Children’s Hospital, University of Helsinki and, HUS Helsinki University Hospital, Helsinki, Finland; 16 Laboratory of Human Genetics of Infectious Diseases, Necker Branch, INSERM, Necker Hospital for Sick Children, Paris, France; 17 Imagine Institute, University of Paris, Paris, France; 18 Department of Pediatrics, Necker Hospital for Sick Children, Paris, France; 19 Howard Hughes Medical Institute, Paris, France; 20 Infectious Diseases, Oulu University Hospital, Oulu, Finland

**Keywords:** STAT3, Pulmonary aspergillosis, Mutation

## Abstract

Signal transducer and activator of transcription 3 (STAT3) plays a key role in leukocytic and non-leukocytic cells. Germ line mutations in STAT3, which are mainly found in the SH2, DNA binding and transactivation domains, can be loss- or gain-of-function (LOF and GOF). *STAT3* N-terminal domain (NTD) mutations are rare, and their biological effects remain incompletely understood. We explored the significance of *STAT3* NTD p.Trp37* variant in a patient with chronic pulmonary aspergillosis and a low Hyper-IgE syndrome (HIES) score. In cell culture models, the expression of full-length p.Trp37* allele showed shorter STAT3 protein expression suggesting a re-initiation (Met99 or Met143). STAT3 activity using luciferase reporter assay showed a twofold-increased activity of the STAT3 p.Trp37* STAT3 protein compared with WT STAT3 at basal level and upon IL-6 stimulation. In contrast, the activity of the short pTrp37* peptide (amino acids 1 to 37) was amorphic but without dominant negative (DN) effect on transcriptional activity or STAT3 Tyr705 phosphorylation. The proteins initiated at Met99 and Met143 were surprisingly hypermorphic. In carriers’ peripheral blood mononuclear cells (PBMCs), both WT and mutated STAT3 mRNA were equally present and the global amount of STAT3 protein was not significantly reduced. In stimulated heterozygous carriers’ PBMCs, however, STAT3 Tyr705 phosphorylation and Th17 were reduced but not completely abolished. This suggests a DN effect of an unknown product of the p.Trp37* allele. Transcriptomics analysis of PBMCs from the index revealed selectively distinct gene expression. We conclude that heterozygosity for the NTD p.Trp37* *STAT3* mutation defines a novel allelic form of STAT3 deficiency, associated with a chronic pulmonary aspergillosis and minor signs of HIES.

**Supplementary Information:**

The online version contains supplementary material available at 10.1007/s10875-025-01867-1.

## Introduction

Dominant negative (DN) loss-of-function (LOF) mutations in signal transducer and activator of transcription 3 (*STAT3*) gene cause hyper IgE syndrome (HIES) [1, 2]. Clinical HIES diagnosis is based on scoring of pneumonia susceptibility, newborn rash, pathological bone fractures, characteristic facies, and high-arched palate [3–5]. *STAT3* gain-of-function (GOF) mutations in turn lead to early-onset autoimmunity and lymphoproliferation [6, 7]. In addition to LOF and GOF, germline “multimorphic” pathologic variants with variable clinical and biological findings and mixed GOF, LOF, and neomorphic effects have recently been reported, also in STAT3 [5, 8, 9]. Heterozygous non-sense STAT3 mutations causing nonsense-mediated decay (NMD) of the affected mRNA have also been described [10, 11] but were not fully investigated in vivo although they have been shown to be dominant by DN effect in vitro [2].

Most autosomal DN *STAT3* LOF pathogenic variants are found in the SH2, DNA binding and transactivation domains [2, 4]. In contrast, a low number of pathogenic germline DN N-terminal domain (NTD) STAT3 variants has been reported. This NTD contain the N-terminal part and the coiled-coil domain and has been involved in the nuclear translocation, and dimerization of STAT3 [7, 12, 13].

We report here the clinical, genetic, and immunological features of a previously healthy young female who presented with an acute onset of bloody sputum caused by chronic pulmonary aspergillosis (CPA) and carried a private STAT3 variant in the NTD of unknown significance.

## Methods

### Genetic Analysis

Exome and targeted Sanger sequencing were completed as previously described in [14]. Whole genome sequencing was performed on Illumina® NovaSeq6000 at the Oslo University Hospital using DNA extracted from whole blood after library prep with Illumina® DNA Prep. DRAGEN 4.1-GATK with reference-genome GRCh38 was used for alignment, annotating and variant calling. An *in-house* curated gene panel containing 862 validated inborn errors immunity (IEI) genes were used for in silico filtering of variants. Splice variants were bioinformatically filtered based on SpliceAI-score [15], and manually checked with prediction tools in Alamut™ Visual Plus.

### Flow Cytometry

For the analysis of intracellular pSTAT3 and pSTAT1, PBMCs were isolated with Ficoll-Plaque gradient centrifugation (GE Healthcare Biosciences AB, Uppsala, Sweden). Isolated PBMC were stored in RPMI-10% FBS-10% DMSO at −140˚C. The cells were thawed, let to rest overnight at 37 ˚C in a cell incubator, washed and stained for 30 min at room temperature with anti-CD4 antibody (Invitrogen, Waltham, USA). After staining the cells were washed twice with supplemented RPMI-2% FBS medium, resuspended (2–5 × 10^6^ cells /mL), and incubated for 30 min at 37 ˚C before the stimulants were added. After 0, 7.5, 15, 30 or 45 min of stimulation with IL-6 (50 ng/mL), IL-21 (10 ng/mL), IL-27 (200 ng/mL) (Peprotech, Neuilly-sur-Seine, France) or IFN-α (10,000U/mL) (Gibco, Waltham, USA) at 37 ˚C, the incubation was stopped with an equal volume of prewarmed 4% formaldehyde for 10 min at 37˚C. After two washes with PBS-2% FBS, chilled Perm Buffer III (BD Biosciences, San Jose, USA) was added on the cells and incubated for 30 min on ice. The samples were washed twice with PBS-2% FBS and stained for pSTAT3 and pSTAT1 with BD Phosflow™ antibodies (BD Biosciences) for 35 min on ice. After washing the cells were acquired using an LSR Fortessa (BD) flow cytometer, and data were analysed using FlowJo software (V10, Tree Star).

For the analysis of total STAT3, the PBMC were thawed and plated in complete RPMI 1640 medium containing 10% FBS (Sigma-Aldrich). The cells were stimulated with IL-6 (50 ng/ml) for 30 min. To detect STAT3, the cells were permeabilized and fixed for 40 min at 4 °C using the Transcription Factor Staining Buffer Set according to the manufacturer’s protocol (Invitrogen). The cells were washed twice with permeabilization buffer and stained for STAT3 (BioLegend) for 40 min at 4 °C. Cells were then washed, acquired, and analysed as before.

### Exon-Trapping Assay

The exon-trapping assay was performed as previously described [2, 16, 17]. STAT3 gDNA was amplified from control fibroblasts. The pair of primers was designed to amplify the genomic region between 200 upstream the exon 2 (5’-cttgagtccaggagttcaaggccac) and 200 nucleotides downstream exon 4 (5’-ccattgggtctgttggattcttttggtg) of *STAT3*. Genomic STAT3 amplified sequence was inserted into the pSPL3 plasmid (Life Technologies). The variant was reintroduced by directed mutagenesis, and the full STAT3 gDNA fragment was Sanger sequenced. Plasmids were used to transfect Cos-7 cells with the X-tremeGENE 9 DNA Transfection Reagent kit (Sigma-Aldrich) according to the manufacturer’s instructions. Cells were harvested 24 h after transfection, and total RNA was extracted as previously described. After cDNA synthesis, PCR was performed with the SD6 (5′-TCTCAGTCACTGGACAACC-3′) and SA2 (5′-ATCTCAGTGGTATTTGTGAGC-3′) primers for pSPL3 plasmids. The amplified cDNAs were inserted into the pCR4-TOPO plasmid vector (Invitrogen). M13 forward (5′-GTAAAACGACGGCCAG-3′) and M13 reverse (5′-CAGGAAACAGCTATGAC-3′) primers were used for amplifications for the analysis of potential splicing. We screened 100 colonies for each variant to analyze splicing.

### NanoString Analysis of Patient PBMCs

NanoString direct mRNA counting was performed with a custom code set as described previously [18]. The list of NanoString targets is in Supplemental Table 2.

### RNA-sequencing

PBMCs from cases and controls were stimulated with IL-6 (30 ng/mL) for 30 min for RNA-sequencing (RNA-Seq). Total RNA was isolated with RNeasy Isolation kit (Qiagen, Hilden, Germany). The RNA-seq libraries preparation and sequencing were performed by BGI Tech Solutions (Hongkong) using the DNBseq platform. Data analysis was performed by BGI Tech Solutions. After filtering and cleaning, the sequencing reads were mapped to a reference genome (hg19_UCSC_20180822) using the HISAT/Bowtie2 tool. Gene expression level was calculated by RNA-seq by Expectation Maximization (RSEM). Differentially expressed genes were screened using PossionDis which is based on the poisson distribution, performed as described at Audic S, et al. [19]. The KEGG database was used to perform pathway enrichment analysis of DEGs between the indexed patient and her control.

### Quantitative RT-PCR

RNA was purified from IL-6-stimulated PBMCs isolated from cases and control samples with an RNeasy kit (Qiagen) according to the manufacturer’s instructions. A SuperScript VILO cDNA Synthesis kit (Invitrogen) was used for complementary DNA (cDNA) synthesis and PROBE FAST ABI Prism 2X qPCR Master Mix (Kapa Biosystems) for the TaqMan Master Mix. Primers for human *FOXP3*, *RORC* and *TBX21* were obtained from Sigma-Aldrich and ThermoFisher Scientific. Gene-specific primers and probes were designed from the Universal ProbeLibrary (Roche Applied Science), and Universal Probe Library probes and custom ordered oligonucleotides are shown in Supplemental Table 3. The relative quantification of gene expression was carried out with Bio-Rad CFX96 Real-Time System. Relative gene expression was calculated using the endogenous control gene EF1alpha to normalise the target gene.

### Luciferase assay

Luciferase assay as performed was previously described [2].

### Transfection of STAT3 p.Tyr37*

STAT3 p.Tyr37* was constructed to MAC-tag-N vector [20] and was transfected to HEK293 cells using calcium phosphate. Two days after transfection, cells were stimulated with or without IL-6 (30 ng/ml, R&D Systems, Minneapolis, MN, USA) for 30 min. Cells were collected for further Western blot detections of tyrosine phosphorylated STAT3 and total STAT3 (Cell Signaling Technology, Danvers, USA).

## Results

### Case Description

**Fig. 1:**
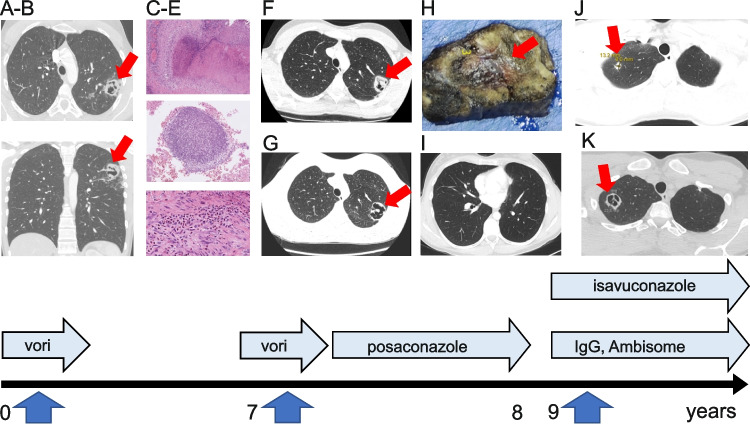
The index presented with bloody sputum at age 22 when her chest CT was consistent with chronic pulmonary aspergillosis (**A, B**). Histology of the lung lesion was consistent with aspergillosis and displayed high numbers of eosinophils (**C-E**). Seven years later, hemoptysis recurred, and chest CT showed a new aspergillosis lesion (**F, G**) which was surgically removed (**H**). One year later, the CT scan appeared unremarkable (**I**). Soon after, several new aspergillosis lesions were found (**J, K**) leading to surgical removal of the largest lesions. Medications (vori, voriconazole; posaconazole; isavuconazole; Ambisome, liposomal amphotericin B; IgG, intravenous IgG substitution) are indicated. Blue solid arrows on the timeline indicate the surgical operations

The index case with mild asthma (patient II:5) had not suffered from infections or features of HIES (NIH HIES score 16, STAT3 score 0) until she developed flu-like symptoms and hemoptysis at age 22. C-reactive protein (CRP) level and sedimentation rate were low. Computed tomography (CT) demonstrated a cavity with a rounded mass in the left lung upper lobe (Fig. 1A-B). Bronchoalveolar lavage and sputum samples were positive for *Aspergillus fumigatus* and negative for other pathogens. Her immunological evaluations were unremarkable although her serum IgE (380–514 IU/L, normal range < 110 IU/L) and serum *Aspergillus* specific IgE (2.03 IU/L; normal < 0.35 IU/L) were somewhat elevated. The cavitating lung lesion was surgically removed. The necrotic tissue samples were positive for *Aspergillus fumigatus* (Fig. 1C-E) and abundant eosinophils. The patient received intravenous voriconazole (200 mg twice daily) followed by oral voriconazole for one month. She had remained asymptomatic with unremarkable chest CT for six years when she developed another episode of cough and bloody sputum. Again, CT scan revealed left upper lobe lesion suggestive of aspergillosis (Fig. 1F-G). The lesion was surgically removed (Fig. 1H). Histology was consistent with aspergillosis and severe tissue necrosis suggesting necrotizing CPA. She received voriconazole followed by oral posaconazole for one year at which point her chest CT was unremarkable (Fig. 1I). Soon after, additional multiple solid lesions were observed in her lung CT (Fig. 1J-K) leading to the need of another surgical treatment. Importantly, the patient did not develop non-infectious pneumatoceles or broncho-pleural fistulae. The patient started to receive oral isavuconazole (serum concentration 3.76 mg/L) and two-week courses of liposomal amphotericin B (Ambisome, 3 mg/kg). Intravenous immunoglobulin G (IgG) substitution was administered due to a slightly low response to the Pneumovax vaccine. Despite these medications, additional pulmonary lesions developed. Asymptomatic family members were carefully investigated, and they were given instructions to avoid mold exposures and to seek for medical attention for prolonged cough. They have access to high quality immunological and clinical care.

### Genetics, STAT3 p.Trp37* Variant Gene Products and STAT3 Phosphorylation

**Fig. 2:**
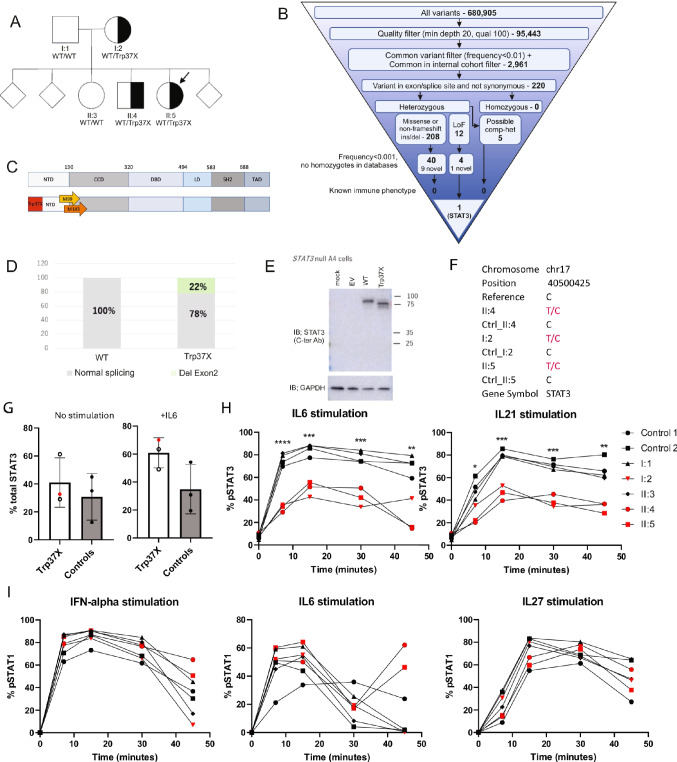
**A** Family segregation of the STAT3 p.Trp37* allele in the kindred. The index (II:5) is marked with a solid arrow. **B** A schematic of the filtering criteria and amounts of variants from whole exome analysis (**C**). Whole exome sequencing revealed a private heterozygous premature stop gained (p.Trp37*) in STAT3 gene. Possible re-initiation sites (methionine 99 and 143) are indicated with arrows. **D** p.Trp37* variant affects the splicing of exon 2 in STAT3. **E** STAT3 immunoblotting on STAT3 deficient A4 cells transfected with WT or p.Trp37* STAT3 variant. p.Trp37* STAT3 variant produced lower MW products compared to WT STAT3, indicating a re-initiation. **F** The STAT3 p.W37X mutation expression from RNA-Seq data. **G** Intracellular STAT3 immunostaining measured by flow cytometry on PBMC from heterozygous Trp37* variant carriers and controls without (left) and with stimulation with IL-6 (right) (**H**). Time course of phospho-STAT3 by intracellular immunostaining in PBMC from heterozygous Trp37* variant carriers, non-carrier relatives, and healthy controls upon IL-6 or IL-21 stimulation. **I** Time course of phospho-STAT1 by intracellular immunostaining in PBMC from Trp37* variant carriers, non-carrier relatives, and healthy controls upon interferon-alpha, IL-6 or IL-21

Whole exome sequencing (WES) revealed a private heterozygous premature termination variant in *STAT3* gene (Chr17:40,500,425 C > T; NM-213662:exon2:c.G110A:p.Trp37*). The STAT3 variant was also found in heterozygosity in her mother (I:2) and her brother (II:4) (Fig. 2A, B and C) who were both asymptomatic, had low HIES score and no history suggestive of HIES, STAT3 GOF or any other immunodeficiency condition. No other genetic variants with potential significance from the patient’s WES have been identified (Supplemental Table 1). Furthermore, no other clinically significant variants were observed in an in-house curated set of known and putative IEI genes based on genome sequencing of the patient’s DNA.

Analysis of the p.Trp37* variant consequence on STAT3 transcript by in vitro exon-trapping in COS-7 cells showed that the variant may lead to alternative splicing events by splicing out the exon 2 and removing the start Methionine (Fig. 2D). Immunoblotting assessment of p.Trp37* STAT3 over-expression in STAT3-negative A4 cells revealed products at a lower molecular weight, consistent with re-initiation at positions M99 and M143, as previously described [21] (Fig. 2E).

The study on PBMCs from I:2, II:4, II:5 individuals by RNA-Seq demonstrated that both alleles (WT: C nucleotide and mutated: T nucleotide) are transcribed (Fig. 2F). However, a slight reduction in the proportion of the p.W37* allele is observed from 40% in individual I:2 to 30% in the index case (II:5) and her brother (II:4). Finally, STAT3 protein expression by immunoblotting or flow cytometry in PBMCs in carrier’s family members were not reduced when compared to healthy controls (Fig. 2G, Supplemental Fig. 1).

We thus assess the activation of STAT3 by measuring the phosphorylation level in all three heterozygous p.Trp37* STAT3 carriers (II:5-index, I:2, II:4) upon IL-6 and IL-21. All displayed 50% reduced STAT3 Tyr705 phosphorylation at different time-points after IL-6 or IL-21 stimulation (Fig. 2H) when compared with healthy controls or family members who are non-carrier of p.Trp37* allele (I:1, II:3). However, STAT1 phosphorylation was at a similar level after IFN-γ, IL-6, or IL-27 stimulation in the p.Trp37* cases and healthy controls (Fig. 2I).

### Peripheral Blood Lymphocytes and Their Subpopulations

Lymphocyte immunophenotyping of patient II:5 was unremarkable. The response to pneumococcal polysaccharide antigens (Pneumovax®) was reduced with adequate responses to only 5 of the 10 tested serotypes [22]. While total CD3^+^CD4^+^ and CD3^+^CD8^+^ T cell counts were normal, effector memory populations were below normal range. Proportion of Th17 in the index case was reduced (9.3%, normal range 19–34%) but not absent. Th1 in turn was above normal range (45%, normal range 16–32%). IL-17 was also lower (0.47) than that observed in healthy control (0.72).

### STAT3 p.Trp37* Transcriptional Activity

**Fig. 3:**
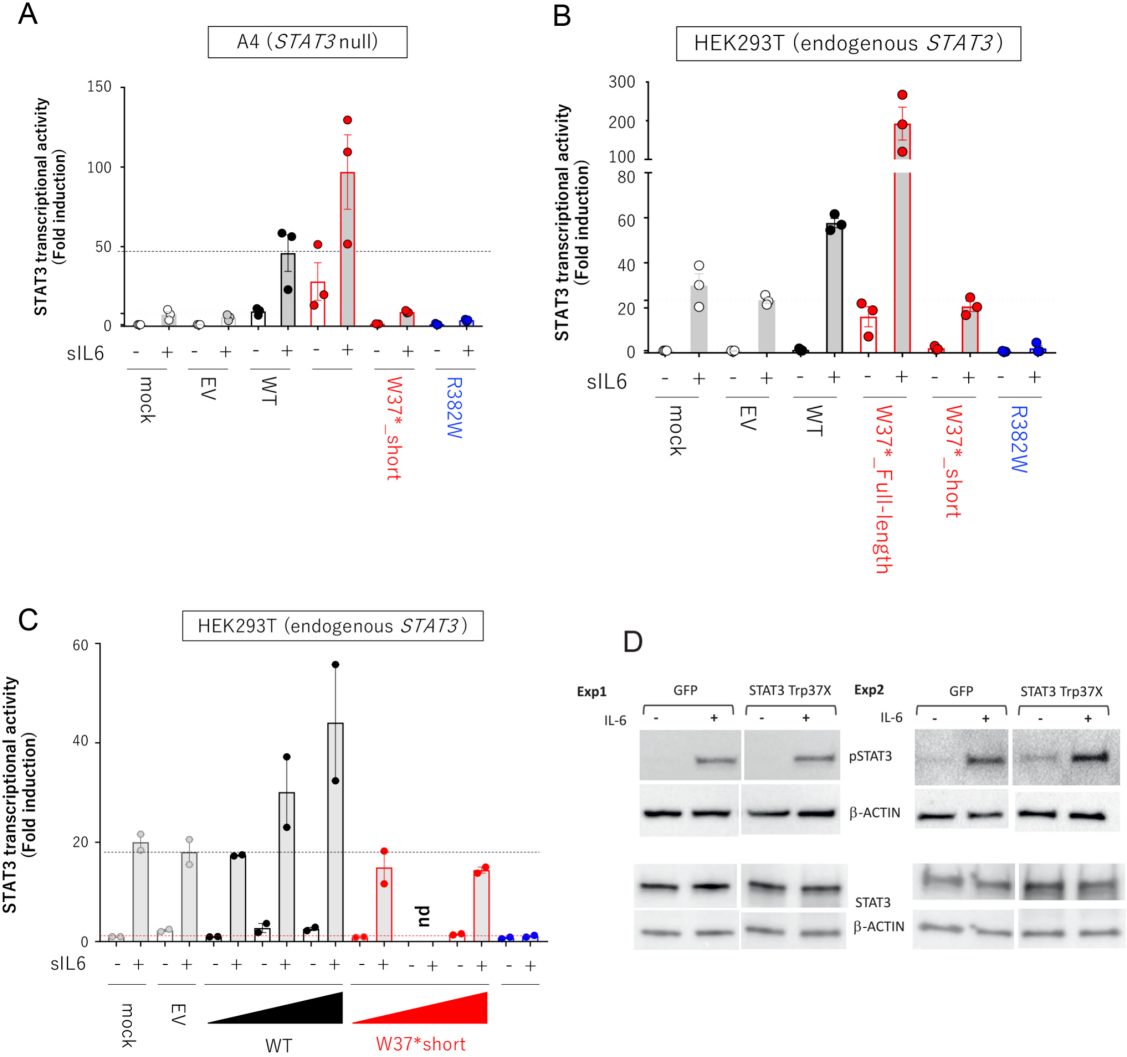
Luciferase activity (fold change) in STAT3 deficient A4 cells in non-transfected (mock), empty vector (EV), wild type STAT3 (WT), whole STAT3 sequence with p.Trp37* (W37*_Full-length), STAT3 N-terminal peptide amino acids 1–37 (W37*_short), or dominant negative STAT3 (Arg382Trp) (**A**). Luciferase activity (fold change) in STAT3 positive HEK293T cells in non-transfected (mock), empty vector (EV), wild type STAT3 (WT), whole STAT3 sequence with p.Trp37* (W37*_Full-length), STAT3 N-terminal peptide amino acids 1–37 (W37*_short), or dominant negative STAT3 (Arg382Trp). The y-axis represents STAT3 transcriptional activity levels normalized against unstimulated activity in EV-transformed cells. Each dot represents a biological replicate obtained from technical replicates. **B** Luciferase activity (fold change) in HEK293T cells with endogenous STAT3 expressing an increasing amount of wild type (WT) or whole STAT3 sequence with p. Trp37X or dominant negative STAT3 (Arg382Trp). **D** HEK293 cells were transfected with GFP and MAC-tag-N vector containing STAT3 p.Trp37*_short using calcium phosphate. Two days after transfection, cells were stimulated with or without IL-6 for 30 min. Cells were collected for further Western blot detections of tyrosine phosphorylated STAT3 and total STAT3. Immunoblots from two independent experiments are shown

In *STAT3* deficient A4 cells, the re-expression of full-length wild type *STAT3* (WT) or full-length cDNA of the p.Trp37*variant (Trp37*_full-length in Fig. 3A) demonstrated increased luciferase activity, which is twice as the control for the Trp37*-full length variant. Instead, expression of a cDNA encoding only the first 37 AA of STAT3 had no detectable effect on activity (Trp37*_short, Fig. 3A and Supplemental Fig. 2). To examine dominant negative effect, WT STAT3 or p.Trp37*_short (Fig. 3B,) in HEK293T which expressed endogenous STAT3 activity were transfected, and luciferase activity determined. A dose-dependent increase in luciferase activity was observed after IL-6 stimulation in HEK293T cells overexpressing WT STAT3. However, increasing amount of STAT3 Trp37*_short in HEK293T cells did not interfere with luciferase activity (Fig. 3C), suggesting that the STAT3 N-terminal 1 to 37 AA peptide does not display DN effect on transcriptional activity, in contrast to STAT3 p.Arg382Trp allele which has been reported to be amorphic and DN (Fig. 3A-C). Finally, HEK293T cells transfected with a control GFP expressing vector or with STAT3 p.Trp37*_short expressed similar levels of phosphorylated STAT3 and total STAT3 after IL-6 stimulation (Fig. 3D).

### Expression Profiles in STAT3 p.Trp37* PBMCs

**Fig. 4:**
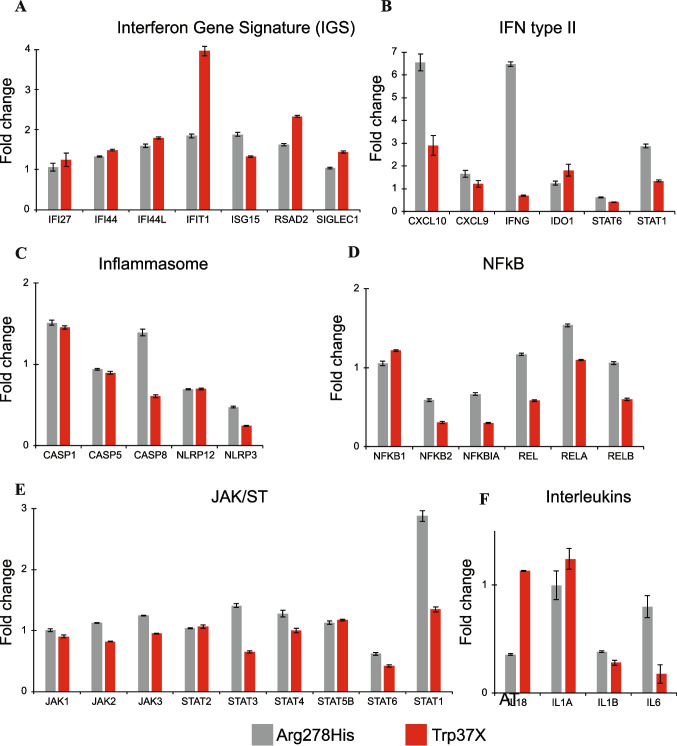
Selected immunological expression profiles of index PBMCs. mRNA fold changes compared to healthy controls in unstimulated index STAT3 p.Trp37* and Arg278His (STAT3 GOF) PBMCs of seven IFN-regulated genes (**A**), type II IFN (**B**), inflammasome (**C**), NF-κB pathway (**D**), JAK/STAT pathway (**E**), and interleukins (**F**) related genes. Mean of three measurements together with SD is presented

Since seemingly conflicting results of reduced phosphorylated STAT3 in vivo and isomorphic transcriptional activity of the p.Trp37* *STAT3* in vitro were observed, we compared the transcriptional expression from unstimulated PBMCs obtained from the index to a patient with confirmed *STAT3* GOF (*STAT3* p.Arg278His) normalised by 2 unstimulated healthy individuals [7]. In *STAT3* p.Arg278His GOF PBMCs, high *CXCL10*, *IFNG* and *STAT1* expression was observed. Similar changes indicative of *STAT3* GOF were not seen in the expression profile of *STAT3* p.Trp37* PBMCs from the index case (Fig. 4). Both these results and the clinical phenotype thus argued against *STAT3* GOF phenotype.

**Fig. 5:**
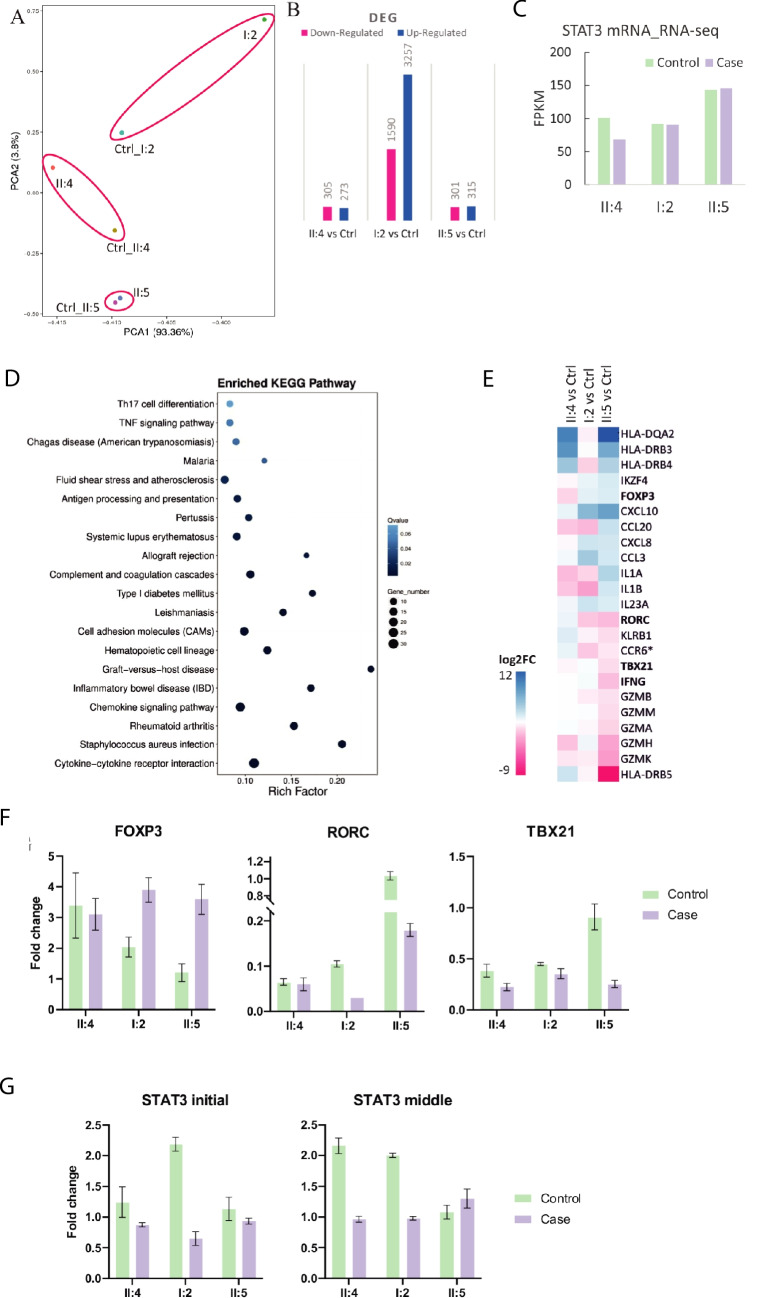
Transcriptomics analysis reveals distinct gene expression profiles in cases with STAT3 p.Trp37* mutation. **A** PCA plot of transcriptomes of IL-6 stimulated PBMCs from three STAT3 p.Trp37* variant positive cases and sex/age matched controls X-axis represents the contributor rate of the first component. Y-axis represents the contributor rate of the second component. Points represent each sample. Ellipses were drawn manually. **B** The number of differentially expressed genes from IL-6 stimulated PBMCs from three STAT3 p. Trp37X mutated cases and sex/age matched controls are shown. **C** STAT3 mRNA expression of the three family members with the STAT3 p.W37X mutation and their controls from RNA-Seq data obtained from IL-6 stimulated PBMCs. **D** KEGG pathway analysis was performed on up-regulated and down-regulated genes in IL-6 stimulated PBMCs from the indexed patient versus her control comparison. The pathways presented in the plot are significantly enriched; The colour indicates the q-value, the lower q-value indicates the more significant enrichment. Point size indicates DEG number. The larger the value of the rich factor, the more significant enrichment. **E** Heatmap showing the log fold change values of selected genes significantly changed in IL-6 stimulated PBMC from patient versus control (**F**). Quantitative RT-PCR detections of *Foxp3*, *RORC* and *TBX21*, key transcription factors for Treg, Th17 and Th1 cells respectively. **G** Quantitative RT-PCR mRNA expression using 5’UTR probe (upstream the W37* variant), and probe in the middle of the transcript (downstream the W37* variant) the p.W37X mutation site respectively, fragments of STAT3. EF1α was used as endogenous control in all the qPCR detections

Stimulated PBMCs from three p.Trp37* variant carriers and their controls underwent RNA-Seq transcriptomic analyses. Principal component analysis (PCA) showed that the transcriptomes of p.Trp37* positive cases were rather similar when compared to matched controls, with minor differences between the expression profiles of the index I:2 and her control (Fig. 5A). Group comparison of the 3 family members versus the 3 controls only generated expression changes of 20 genes (Supplemental Table 4). Among them, a significant up-regulation of *CXCL9* in the 3 carriers has been found, a gene previously reported as upregulated in T-cell from CD4-conditional STAT3-deficient mice [23]. Since the other two family members are healthy, and they do not suffer from infections or HIES phenotype, we focused on comparison of expression profiles between the index patient and her control. Of the 15,596 genes detected in IL-6 stimulated PBMCs from the index and her control sample, expression of 301 genes was reduced in IL-6 stimulated p.Trp37* PBMCs and expression of 315 genes was enhanced when compared with the control, suggesting the possibility of neomorphic effects (Fig. 5B, Supplemental Table 5). Importantly, as shown in Fig. 2F, the RNA-Seq results performed from primary patient cells have confirmed the presence of the reported variant. Notably, the RNA-Seq data showed no changes of total STAT3 mRNA expression level in the three heterozygous p.Trp37* variant family carriers versus controls (Fig. 5C). Interestingly, the Th17 differentiation pathway was dissimilar (Fig. 5D), with reduced expression of *RORC*, *TBX21*, *IFNG* and *HLA-DRB5* in the patient’s sample (Fig. 5E, 5F) [24]. Expression of 10 selected genes, including *FOXP3* and *IKAROS Family Zinc Finger 4* (*IKZF4*) was significantly upregulated in the patient’s sample compared with her control (Fig. 5D-F). qPCR further confirmed the increased expression of *FOXP3* and reduced *RORC* and *TBX21* in the index patient’s IL-6 stimulated PBMCs [25]. Finally, we explored whether the variant could create a new allele by quantifying the *STAT3* cDNA expression using probes targeting the 5’ (exon 2) or the middle (exon 14) of *STAT3* by qPCR. As shown in Fig. 5G, no difference using both probes were observed on the PBMCs from the index or compared to her control.

## Discussion

Chronic pulmonary aspergillosis (CPA) is a rare condition that can be found in association with lung diseases such as tuberculosis, non-tuberculous mycobacterial infection, or allergic bronchopulmonary aspergillosis [26]. In addition to severely immunosuppressed individuals, pulmonary aspergillosis may also affect critically ill patients especially among the elderly [27]. However, the young index patient described here had no history of lung disease, infection susceptibility or immunosuppressive condition. Instead, the CPA in this case associates with novel and rare STAT3 p.Trp37* NTD premature termination mutation; only two individuals with similar STAT3 NTD variant have been identified in gnomAD genetic database (p.Gln20Ter, p.Glu39Ter; allele frequency 1.59 × 10^–6^). Clinical presentation or immunological findings associated with such NTD variants are not well understood. In contrast to STAT3 LOF or GOF variants, incomplete clinical penetrance and lack of classical phenotype are evident in this family [4]. Natarajan et al. reported a case of STAT3 haploinsufficiency with reduced amount of STAT3 caused by a splice-site mutation and nonsense-mediated decay (NMD) of the affected allele [10]. In contrast, the p.Trp37* mutant mRNA species were present, and the total STAT3 protein level was not reduced in the PBMCs collected from the patient. In summary, the genetics and clinical presentation in the case described here are suggestive of unique phenotype.

Disruption of the STAT3 NTD caused by p.Trp37* allele may lead to several biological consequences. Obviously, the mutation can be expected to disturb the STAT3 NTD cooperative DNA binding, nuclear translocation, and protein–protein interactions including dimerization of STAT3 [13, 28]. Although the p.Trp37* disrupts the NTD and the short 37 AA peptide is not DN in experimental models [12], the premature termination leads in an in vitro assay to re-initiation products with higher biological activity. Despite the high activity in vitro, the patient’s clinical and immunological phenotype differs from STAT3 GOF. While the p.Trp37* allele does not have DN effect on transcriptional activity in vitro cell culture model, this combination of alleles disturbs the WT STAT3 Y705 phosphorylation and Th17 cell maturation, mimicking DN STAT3 deficiency. Although we are not able to define the exact biological mechanism, the STAT3 p.Trp37* generates a novel combination of disturbed STAT3 biology.

In summary, we report a patient with chronic pulmonary aspergillosis and a heterozygous STAT3 NTD premature termination p.Trp37* LOF allele. She presents with a novel combination of mixed biological and clinical features. Clinical and biological information involving the STAT3 NTD LOF variants in literature is limited; the case presented here deviates from previously reported DN STAT3 HIES, or STAT3 GOF cases. Incomplete clinical penetrance in family members suggest that external causes such as exposure to an aggressive *Aspergillus* strain or additional genetic components may have contributed to this condition.

## Supplementary Information

Below is the link to the electronic supplementary material.

ESM 1 (XLSX 48.8 KB)

ESM 2 (PDF 186 KB)

## Data Availability

Available upon reasonable request to the corresponding authors.
