# Supplementary material for: Pulmonary Aspergillosis and Low HIES Score in a Family with STAT3 N-Terminal Domain Mutation
Source: J Clin Immunol. 2025 Feb 10;45(1):73. doi: 10.1007/s10875-025-01867-1 (PMC11811237; doi:10.1007/s10875-025-01867-1)

Supplemental Fig. 1

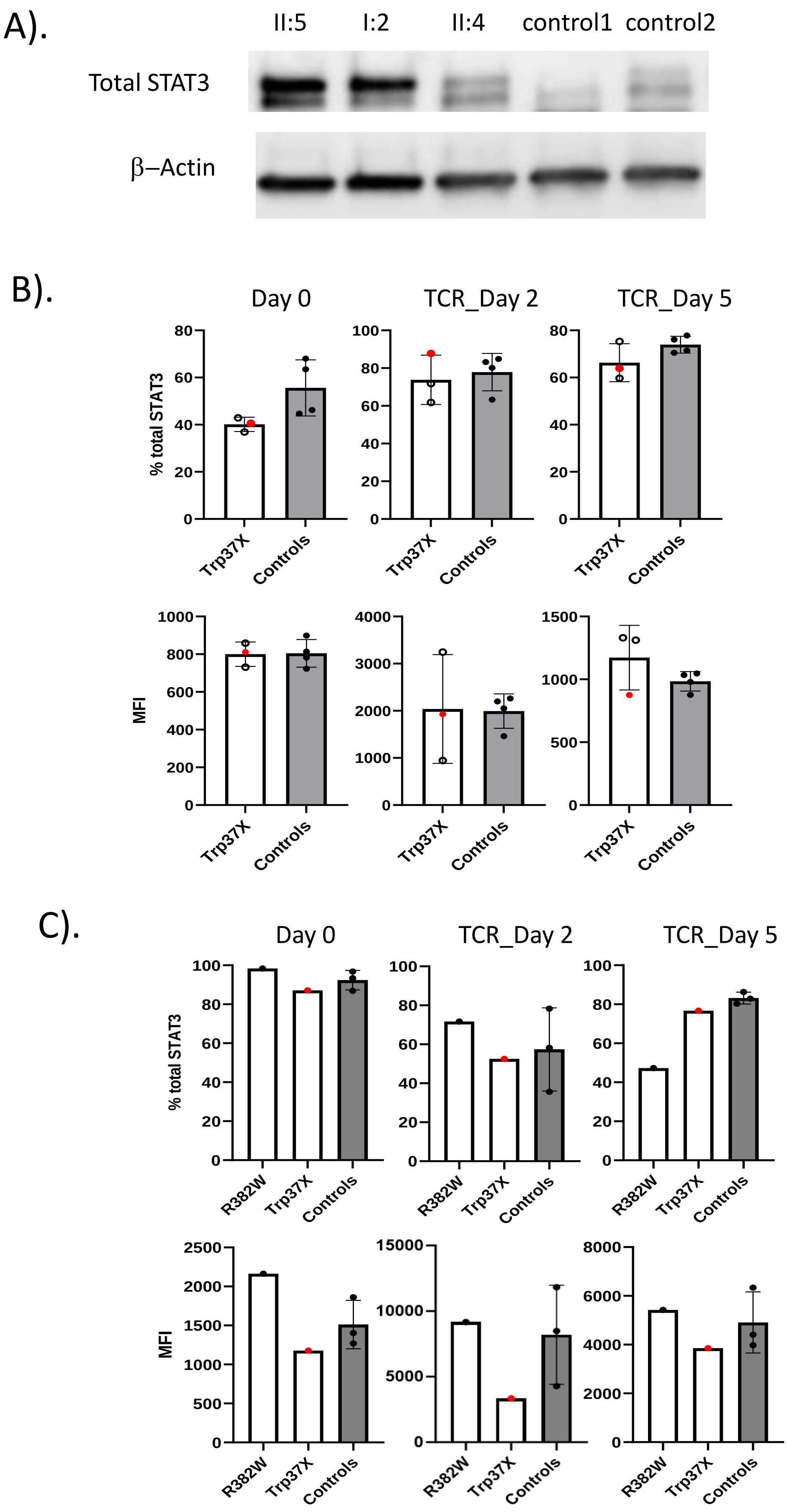

S.Fig 1. A). Immunoblotting with STAT3 antibody on total protein extracts from PBMCs of heterozygous Trp37* carriers, and healthy controls.
B). Flow cytometry detection of total STAT3 after stimulation with anti-CD3/anti-CD28 (Day 2) or IL-2 (Day 5) in p. Trp37* variant positive carriers (index in red) and healthy controls shown as total percentage (upper panel) and expression level (lower panel. GMFI, geometric mean fluorescence intensity)
C). Flow cytometry detection of total STAT3 after stimulation with anti-CD3/anti-CD28 (Day 2) or IL-2 (Day 5) in patient with dominant negative STAT3 (R382W), p. Trp37* variant positive index patient, and healthy controls shown as total percentage (upper panel) and expression level (lower panel. GMFI, geometric mean fluorescence intensity)

Supplemental Fig. 2

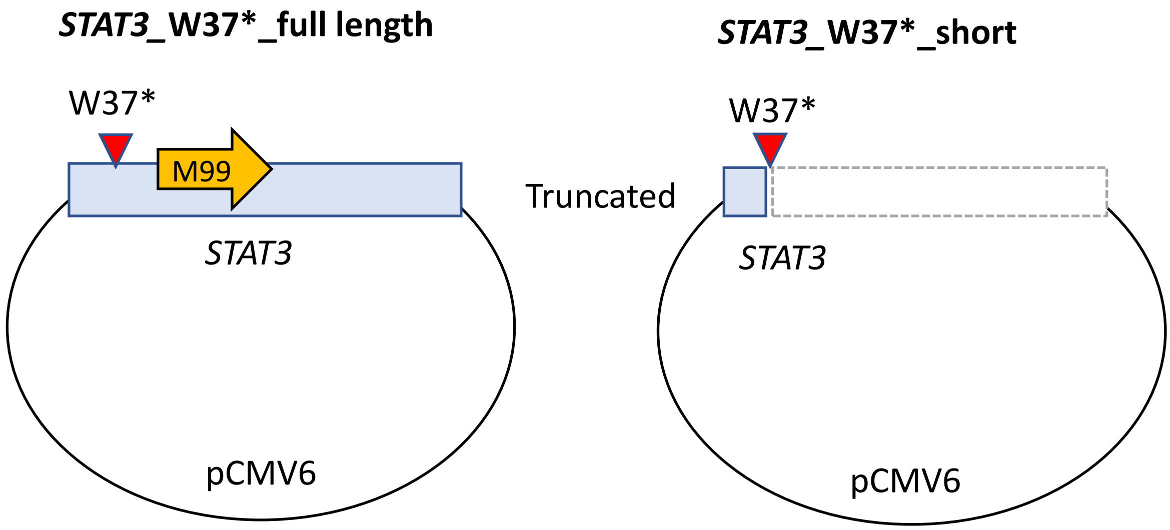

Supplement: Supplementary file 2 — (PDF 186 KB) [file 10875_2025_1867_MOESM2_ESM.pdf]
